# Identifying risk patterns in older adults with atrial fibrillation by hierarchical cluster analysis: A retrospective approach based on the risk probability for clinical events

**DOI:** 10.1016/j.ijcha.2021.100883

**Published:** 2021-09-28

**Authors:** Shinya Suzuki, Takeshi Yamashita, Takayuki Otsuka, Takuto Arita, Naoharu Yagi, Mikio Kishi, Hiroaki Semba, Hiroto Kano, Shunsuke Matsuno, Yuko Kato, Tokuhisa Uejima, Yuji Oikawa, Minoru Matsuhama, Mitsuru Iida, Tatsuya Inoue, Junji Yajima

**Affiliations:** a Department of Cardiovascular Medicine, The Cardiovascular Institute, Tokyo, Japan; b Department of Cardiovascular Surgery, The Cardiovascular Institute, Tokyo, Japan

**Keywords:** Atrial fibrillation, Older adults, Cluster analysis, Anticoagulation, Rhythm control, Rate control

## Abstract

**Background:**

Older adults with atrial fibrillation (AF) have highly diverse risk levels for mortality, heart failure (HF), thromboembolism (TE), and major bleeding (MB), thus an integrated risk-pattern algorithm is warranted.

**Methods:**

We analyzed 573 AF patients aged ≥ 75 years from our single-center cohort (Shinken Database 2010–2018). The 3-year risk scores (risk probability) for mortality (M-score), HF (HF-score), TE (TE-score), and MB (MB-score) were estimated for each patient by logistic regression analysis. Using the four risk scores, cluster analysis was performed with Ward’s linkage hierarchical algorithm.

**Results:**

Three clusters were identified: Clusters 1 (n = 429, 74%), 2 (n = 24, 5%), and 3 (n = 120, 21%). The clusters were characterized as standard risk (Cluster 1), high TE- and MB-risk (Cluster 2), and high M- and HF-risk (Cluster 3). Oral anticoagulants were prescribed for over 80% of the patients in each cluster. Catheter ablation for AF was performed only in Cluster 1 (8.9%). Compared with Cluster 1, Cluster 2 was more closely associated with males, asymptomatic AF, history of cerebral infarction or transient ischemic attack, history of intracranial hemorrhage, high HAS-BLED score (≥3), and low body mass index (<18.0 kg/m^2^). Cluster 3 was more closely associated with old age, heart failure, and low estimated creatinine clearance (<30 mL/min).

**Conclusion:**

The cluster analysis identified those at a high risk for all-cause death and HF or a high risk for TE and MB and could support decision making in older adults with AF.

## Highlights

• In older AF patients, representative AF-related outcomes compete, causing difficulty in decision making.
• We proposed cluster analysis using risk probability for four AF-related outcomes.
• Older adults with AF were classified into 3 clusters.
• The clusters could possibly identify older adults with AF with good/poor responses to AF-related treatment.

## 1 Introduction

Atrial fibrillation (AF) is one of the most common arrhythmias associated with increased mortality and morbidities such as thromboembolism (TE) and heart failure (HF). Recently, older adults and very old adults with AF have numerically increased, and the ratio of AF in these age groups is projected to increase in the near future [1]. Given the increased risk of TE with aging, anticoagulation therapy is needed, but the risk of bleeding also increases with aging, leading to the underuse or underdosing of anticoagulants [2], [3]. The issue of patient age also exists in the prevention of HF. As the chance of the coexistence of HF and AF increases with age, there is an increased need to suppress AF to prevent HF, which is difficult in older adults and patients with HF [4]. Furthermore, the benefit of catheter ablation to prevent HF in AF patients tends to decrease in older adults [5].

The two fundamentals of AF treatment, anticoagulation therapy and AF rhythm control, including catheter ablation, can potentially provide tremendous benefits to older adults with AF if patient selection is successfully performed. Although older adults with AF are generally regarded as being vulnerable and therefore at a high risk for various complications of AF therapy, they have a great heterogeneity [6], which triggers large variations in their treatment responses and clinical outcomes.

Cluster analyses have been shown to facilitate the novel categorization of populations with a mixture of complex characteristics. For heterogenous AF patients, such classifications would be informative. Multiple attempts to use cluster analyses for AF patients have already been reported [7], [8]. Using dozens of baseline parameters, these clarified that approximately half of AF patients, including young and paroxysmal AF patients, are at a low risk for cardiovascular or neurological adverse events, whilst a paucity of patients, including older adults and atherosclerotic patients, are at a high risk for these events. However, a different approach may be warranted in the stratification of older adults with AF to aid decision making in daily clinical practice. Although cardiovascular or neurological adverse events are frequent in older adults, antithrombotic therapy increases the risk of bleeding in this population. Moreover, a high incidence rate of mortality (mostly, non-cardiovascular) masks the impact of both cardiovascular or neurological adverse events and bleeding (competing risks). Given the complex situations and various clinical outcomes associated with older adults with AF, risk stratifications based on baseline characteristics may be inadequate. In contrast, understanding the incidence patterns of various clinical outcomes would offer more information. For this purpose, the computed risk probabilities for clinical outcomes [9], which represent the potential risks for each patient, could contribute to a more integrated categorization of the increasing number of older adults with AF.

In the present study, we performed cluster analysis using the computed risk probability for representative AF-related outcomes, including all-cause death, HF events, TE events, and major bleeding (MB) events, to obtain a novel framework for categorizing older adults with AF.

## 2 Material and methods

### 2.1 Ethics and informed consent

This study was performed in accordance with the Declaration of Helsinki (revised in 2013) and Ethical Guidelines for Medical and Health Research Involving Human Subjects (Public Notice of the Ministry of Education, Culture, Sports, Science and Technology, and the Ministry of Health, Labour, and Welfare, Japan; issued in 2017). Written informed

consent was obtained from all participants. The study protocol was reviewed by the Institutional Review Board of the Cardiovascular Institute.

### 2.2 Study population

The Shinken Database [10] includes information on all patients that newly visited the Cardiovascular Institute, Tokyo, Japan. This single hospital-based database was established in June 2004 to investigate the prevalences and prognoses of various types of cardiovascular diseases (CVDs). To investigate the new appearance of CVDs, patients who visited our hospital but were not diagnosed as having CVDs at baseline were also included in the cohort. The patients are continually registered in the database annually, and the registration is still ongoing (up to March 2018, 24,668 patients were registered with follow-up data). Foreign travelers and patients with active cancer were excluded because of the difficulty with evaluating long-term follow-up. The hospital is a specialized cardiology hospital in an urban area of Tokyo, Japan. The patients seen were local residents that had been referred from other clinics for the treatment of CVDs. The attending physicians were all cardiologists or cardiothoracic surgeons.

In the present study, out of the 24,668 patients in the Shinken Database (between June 2004 and March 2018), 12,891 patients registered between February 2010 and March 2018 were included. Among them, 3,017 patients were diagnosed as having AF at the initial visit. Of these, 573 AF patients aged 75 or over were the target population in the present study. Details of the data collected at the initial visit and patient follow-ups are explained in the supplementary document (See *S1.1. Data collection at initial visit* and *S1.2 Patient follow-up*).

### 2.3 AF treatment

In the present study, the statuses of the AF-related treatments were compared among the determined clusters. The AF-related treatments included (1) oral anticoagulants; (2) antiarrhythmic drugs for rhythm control (class I and III); (3) antiarrhythmic drugs for rate control (class II and IV, and digoxin); and (4) non-pharmacotherapies, including direct cardioversion, catheter ablation for AF, and pacemaker implantation.

### 2.4 Evaluation and statistical analysis

Statistical analyses were carried out using SPSS version 27.0 (IBM Corp., Armonk, NY, USA). In all analyses, *P* < 0.05 was taken to indicate statistical significance. Categorical data are presented as the number (%). Continuous data are presented as the mean ± SD or median (inter-quartiles) for normally and non-normally distributed data, respectively.

#### 2.4.1 Patient categorization

The study participants were categorized via the following steps.

##### 2.4.1.1 Computing risk scores for patient outcomes by multivariable logistic regression analysis

Risk scores for all-cause death (M-score; M derived from mortality), HF events (HF-score), TE events (TE-score), and MB events (MB-score) were computed as incident probabilities by multivariable logistic regression analysis. The following parameters were forcedly entered into the multivariable model: age (continuous variable), sex (male: 1, female: 0), BMI (category; ≥25 kg/m^2^: 0, <25 and ≥18 kg/m^2^: 1, <18 kg/m^2^: 2), systolic blood pressure (category; ≥150 mmHg: 0, <150 and ≥ 100 mmHg: 1, <100 mmHg: 2), serum albumin (category; ≥4.5 g/dL: 0, <4.5 and ≥ 3.5 g/dL: 1, <3.5 g/dL: 2), hemoglobin (category; ≥13 g/dL: 0, <13 and ≥ 11 g/dL: 1, <11 g/dL: 2), eCCr (category; ≥50 mL/min: 0, <50 and ≥ 30 mL/min: 1, <30 mL/min: 2), Charlson’s comorbidity index (continuous variable), incidence of a fall within 3 years after the initial visit, ischemic heart disease, valvular heart disease, cardiomyopathy (dilated, hypertrophic, and others), heart failure, hypertension, dyslipidemia, diabetes mellitus, hyperuricemia, history of ischemic stroke or transient ischemic attack (TIA), history of intracranial hemorrhage, chronic obstructive pulmonary disease, and maintenance dialysis.

##### 2.4.1.2. Hierarchical cluster analysis

2.4.1.2

Using the risk scores for the four outcomes (M-score, HF-score, TE-score, and MB-score), cluster analysis was performed with Ward’s linkage hierarchical algorithm [11]. The number of clusters was set at the maximum pseudo F statistic [12].

#### 2.4.2 Comparison of the clusters

The distribution of the four risk scores (M-score, HF-score, TE-score, and MB-score), patient characteristics, and the statuses of AF treatments were compared among the clusters. The differences among the clusters for the categorical variables were tested by chi-squared test, and those for the continuous variables with parametric and nonparametric distribution were tested by one-way analysis of variance and the Jonckheere-Terpstra test, respectively.

## 3 Results

### 3.1 Patient categorization

#### 3.1.1 Multivariable logistic regression analysis

**Supplementary Fig. 1:**
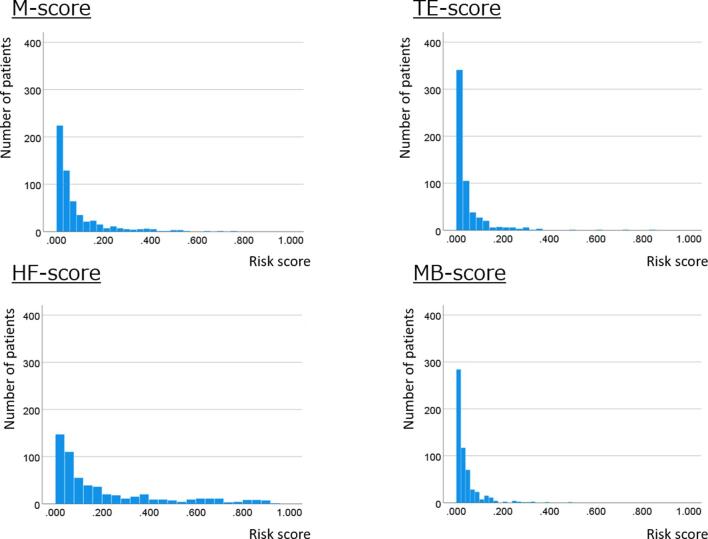
Distribution of risk scores. M-score, HF-score, TE-score, and MB-score indicate the risk probabilities for all-cause death, heart failure events, thromboembolic events, and major bleeding events and were computed by multiple logistic regression analysis

The number of clinical outcomes was 47 (8.2%) for all-cause death, 81 (14.1%) for HF events, 16 (2.8%) for TE events, and 22 (3.8%) for MB events. The results of the multivariable logistic regression analysis are shown in Supplementary Table 1. The distributions of the risk scores are presented in Supplementary Table 2 and Supplementary Fig. 1.

#### 3.1.2 Hierarchical cluster analysis

**Fig. 1:**
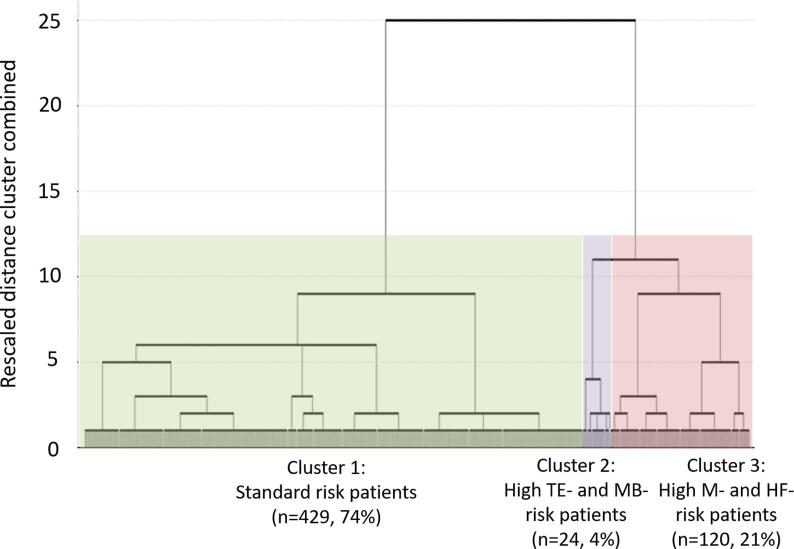
Dendrogram of hierarchical cluster analysis.

Using the four risk scores (M-score, HF-score, TE-score, and MB-score), hierarchical cluster analysis was performed. On the basis of pseudo F statistics, the appropriate number of clusters was determined to be three. The dendrogram is shown in Fig. 1. The final clusters were Cluster 1 (n = 429, 74.9%), Cluster 2 (n = 24, 4.2%), and Cluster 3 (n = 120, 20.9%).

### 3.2 Characteristics of the three clusters in the older adults with AF

#### 3.2.1 Patterns of the risk scores

**Fig. 2:**
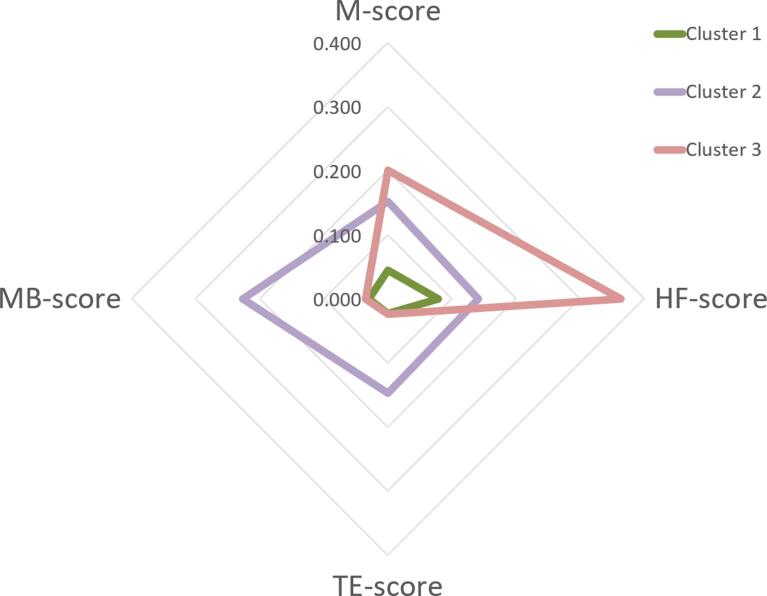
Patterns of risk scores for the three clusters.

The mean M-score, HF-score, TE-score, and MB-score for the three clusters are plotted in Fig. 2. The mean value ± SD (min/max) of the M-score, HF-score, TE-score, and MB-score are listed in Supplemental Table 2. Based on the patterns of the risk scores, the clusters were roughly characterized as Cluster 1, with standard risks for all four outcomes; Cluster 2, with high TE- and MB-risks; and Cluster 3, with high M- and HF-risks.

#### 3.2.2 Patient characteristics

**Fig. 3:**
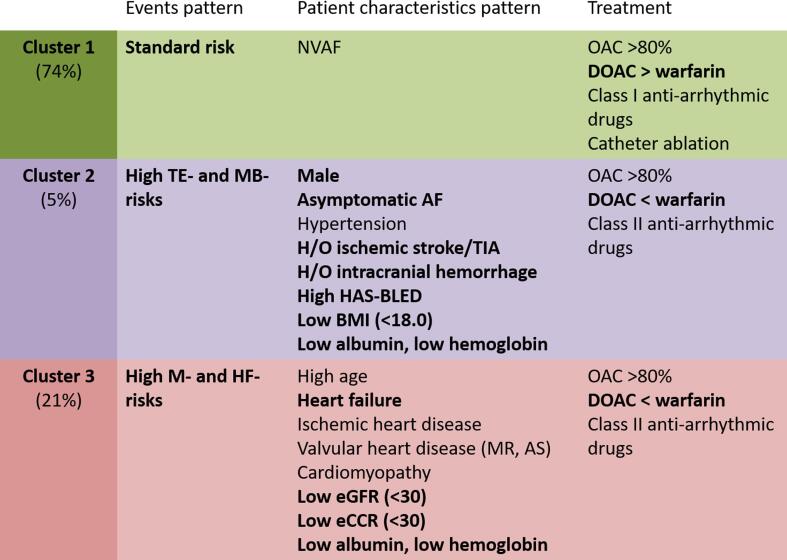
Characteristics of the three clusters.

The clinical characteristics of the patients within each cluster are listed in Table 1, Table 2, and a summary is provided in Fig. 3. The mean age was the highest in Cluster 3 (84.1 ± 4.7 years) compared with Cluster 1 (79.4 ± 3.6 years) and Cluster 2 (78.3 ± 3.1 years) (P < 0.001). Males were more dominant in Cluster 2 (83.3%) compared with Cluster 1 (56.2%) and Cluster 3 (52.5%) (P = 0.020). The prevalence of paroxysmal AF was generally similar among the clusters (Cluster 1, 58.0%; Cluster 2, 50.0%; Cluster 46.7%; P = 0.415), while the prevalence of asymptomatic AF was higher in Cluster 2 (33.3%) than the other clusters (Cluster 1, 15.9%; Cluster 2, 12.5%; P = 0.039).

**Table 1:** Patient characteristics.

	Total	Cluster 1	Cluster 2	Cluster 3	P value
		Standard risk	High TE- and MB- risk	High M- and HF- risk	
	(n = 573)	(n = 429)	(n = 24)	(n = 120)	
Age, years old	80.4 ± 4.3	79.4 ± 3.6	78.3 ± 3.1	84.1 ± 4.7	<0.001
Age (category, years old)					<0.001
75–79	280 (48.9)	246 (57.3)	16 (66.7)	18 (15)	
80–84	198 (34.6)	138 (32.2)	7 (29.2)	53 (44.2)	
≥85	95 (16.6)	45 (10.5)	1 (4.2)	49 (40.8)	
Male	324 (56.5)	241 (56.2)	20 (83.3)	63 (52.5)	0.020
Types of AF					0.415
Paroxysmal AF	317 (55.3)	249 (58.0)	12 (50.0)	56 (46.7)	
Non-paroxysmal AF	256 (44.7)	180 (42.0)	12 (50.0)	64 (53.3)	
Asymptomatic AF	91 (15.9)	68 (15.9)	8 (33.3)	15 (12.5)	0.039
Non-valvular AF	511 (89.2)	397 (92.5)	20 (83.3)	94 (78.3)	<0.001
Ischemic heart disease	82 (14.3)	55 (12.8)	2 (8.3)	25 (20.8)	0.060
Valvular heart disease	240 (41.9)	135 (31.5)	17 (70.8)	88 (73.3)	<0.001
Mitral stenosis	6 (1.0)	5 (1.2)	0 (0.0)	1 (0.8)	0.833
Mitral regurgitation	92 (16.1)	48 (11.2)	4 (16.7)	40 (33.3)	<0.001
Aortic stenosis	82 (14.3)	45 (10.5)	4 (16.7)	33 (27.5)	<0.001
Aortic regurgitation	37 (6.5)	19 (4.4)	3 (12.5)	15 (12.5)	0.003
Tricuspid regurgitation	120 (20.9)	69 (16.1)	11 (45.8)	40 (33.3)	<0.001
History of valvular surgery	59 (10.3)	30 (7.0)	4 (16.7)	25 (20.8)	<0.001
Cardiomyopathy	56 (9.8)	18 (4.2)	2 (8.3)	36 (30.0)	<0.001
Dilated cardiomyopathy	14 (2.4)	2 (0.5)	0 (0.0)	12 (10.0)	<0.001
Hypertrophic cardiomyopathy	33 (5.8)	13 (3.0)	2 (8.3)	18 (15.0)	<0.001
Others	9 (1.6)	3 (0.7)	0 (0.0)	6 (5.0)	0.003
Heart failure (NYHA ≥ II)	173 (30.2)	96 (22.4)	1 (4.2)	76 (63.3)	<0.001
Hypertension	401 (70.0)	288 (67.1)	22 (91.7)	91 (75.8)	0.011
Dyslipidemia	209 (36.5)	160 (37.3)	8 (33.3)	41 (34.2)	0.778
Diabetes mellitus	144 (25.1)	102 (23.8)	4 (16.7)	38 (31.7)	0.132
Hyperuricemia	168 (29.3)	109 (25.4)	4 (16.7)	55 (45.8)	<0.001
History of cerebral infarction or transient ischemic attack	56 (9.8)	29 (6.8)	10 (41.7)	17 (14.2)	<0.001
History of intracranial hemorrhage	8 (1.4)	3 (0.7)	4 (16.7)	1 (0.8)	<0.001
History of bleeding requiring hospitalization	9 (1.6)	6 (1.4)	1 (4.2)	2 (1.7)	0.567
Chronic obstructive pulmonary disease	12 (2.1)	4 (0.9)	0 (0.0)	8 (6.7)	<0.001
Maintenance dialysis	12 (2.1)	4 (0.9)	2 (8.3)	6 (5.0)	0.002
CHADS2 score*	2 (2–3)	2 (2–3)	3 (2–4)	3 (2–4)	<0.001
CHADS2 score ≥ 2	471 (82.2)	338 (78.8)	23 (95.8)	110 (91.7)	0.001
CHA2DS2-VASc score*	4 (3–5)	4 (3–4)	4 (3–5)	5 (4–5)	<0.001
CHA2DS2-VASc score ≥ 3	508 (88.7)	370 (86.2)	23 (95.8)	115 (95.8)	0.007
HAS-BLED score*	2 (2–3)	2 (2–3)	3 (2–5)	3 (2–4)	<0.001
HAS-BLED score ≥ 3	232 (40.5)	149 (34.7)	16 (66.7)	67 (55.8)	<0.001
Charlson's comorbidity index (updated in 2011) *	2 (0–2)	1 (0–2)	2 (1–2)	2 (2–3)	<0.001
Dementia	17 (3.0)	12 (2.8)	0 (0.0)	5 (4.2)	0.502
History of fall or fracture at baseline	20 (3.5)	11 (2.6)	0 (0.0)	9 (7.5)	0.021
Fall or fracture during the observation period	46 (8.0)	17 (4.0)	4 (16.7)	25 (20.8)	<0.001

TE- and MB-, thromboembolism and major bleeding; M- and HF-, mortality and heart failure; AF, atrial fibrillation; NYHA, New York Heart Association functional classification.

* CHADS2 score, CHA2DS2-VASc score, HASBLED score, and Charlson's comorbidity index are presented as median (inter-quartiles range).

**Table 2:** Laboratory data.

	Total	Cluster 1	Cluster 2	Cluster 3	P value
		Standard risk	High TE- and MB- risk	High M- and HF- risk	
	(n = 573)	(n = 429)	(n = 24)	(n = 120)	
Systolic blood pressure, mmHg					0.156
≥150	81 (14.1)	53 (12.4)	6 (25.0)	22 (18.3)	
100–149	471 (82.2)	358 (83.4)	17 (70.8)	96 (80.0)	
<100	21 (3.7)	18 (4.2)	1 (4.2)	2 (1.7)	
Body mass index, kg/m^2^					0.071
≥25	135 (23.6)	97 (22.6)	6 (25.0)	32 (26.7)	
18.0–24.9	397 (69.3)	305 (71.1)	13 (54.2)	79 (65.8)	
<18.0	41 (7.2)	27 (6.3)	5 (20.8)	9 (7.5)	
Albumin, g/dL					<0.001
≥4.5	45 (7.9)	45 (10.5)	0 (0.0)	0 (0.0)	
3.5–4.4	464 (81.0)	362 (84.4)	15 (62.5)	87 (72.5)	
<3.5	64 (11.2)	22 (5.1)	9 (37.5)	33 (27.5)	
Hemoglobin, g/dL					<0.001
≥13.0	246 (42.9)	203 (47.3)	8 (33.3)	35 (29.2)	
11.0–12.9	249 (43.5)	194 (45.2)	9 (37.5)	46 (38.3)	
<11.0	78 (13.6)	32 (7.5)	7 (29.2)	39 (32.5)	
Estimated glomerular filtration rate, mL/min/1.73 m^2^					<0.001
≥60	208 (36.3)	159 (37.1)	15 (62.5)	34 (28.3)	
30–59	232 (40.5)	192 (44.8)	5 (20.8)	35 (29.2)	
<30	133 (23.2)	78 (18.2)	4 (16.7)	51 (42.5)	
Estimated creatinine clearance, mL/min					<0.001
≥50	247 (43.1)	205 (47.8)	18 (75.0)	24 (20.0)	
30–49	270 (47.1)	194 (45.2)	4 (16.7)	72 (60.0)	
<30	56 (9.8)	30 (7.0)	2 (8.3)	24 (20.0)	

TE- and MB-, thromboembolism and major bleeding; M- and HF-, mortality and heart failure.

The prevalence of HF was extremely high in Cluster 3 (63.3%) compared with the other clusters (Cluster 1, 22.4%; Cluster 2, 4.2%; P < 0.001). The prevalence of hypertension, history of ischemic stroke or TIA, and history of intracranial hemorrhage were very much higher in Cluster 2 (91.7%, 41.7%, and 16.7%, respectively) than the other clusters (67.1%, 6.8%, and 0.7% for Cluster 1; 75.8%, 14.2%, and 0.8% for Cluster 3; P = 0.011, <0.001, and <0.001, respectively). The proportion of those with a CHADS2 score ≥ 2 was higher in both Cluster 2 (95.8%) and Cluster 3 (91.7%) compared with Cluster 1 (78.8%, P = 0.001). The proportion of those with HAS-BLED score ≥ 3 was also higher in both Cluster 2 (66.7%) and Cluster 3 (55.8%) compared with Cluster 1 (34.7%, P < 0.001).

#### 3.2.3 AF-related treatment

The AF-related treatments, including oral anticoagulants, antiarrhythmic drugs, and non-pharmacological treatments, are presented in Table 3, with a summary in Fig. 3. The prescription rate of oral anticoagulants was similar among the three clusters (Clusters 1, 81.4%; 2, 87.5%; and 3, 81.7%; P = 0.751). The prescription rate of warfarin was higher in both Cluster 2 (62.5%) and Cluster 3 (53.3%) compared with Cluster 1 (34.0%; P < 0.001), whereas a low percentage time in the therapeutic range (TTR; <60%) was observed more frequently in Clusters 2 (29.2%) and 3 (28.3%) than Cluster 1 (15.9%; P < 0.001). The prescription rate of direct oral anticoagulants (DOACs) was higher in Cluster 1 (47.1%) compared with the other clusters (29.2% for Cluster 2; 28.3% for Cluster 3; P < 0.001).

**Table 3:** Treatment.

	Total	Cluster 1	Cluster 2	Cluster 3	P value
		Standard risk	High TE- and MB- risk	High M- andHF- risk	
	(n = 573)	(n = 429)	(n = 24)	(n = 120)	
Number of drugs	7.9 ± 6.9	6.9 ± 6.1	10.9 ± 10.0	10.9 ± 7.8	<0.001
Oral anticoagulants	468 (81.7)	349 (81.4)	21 (87.5)	98 (81.7)	0.751
Warfarin	225 (39.3)	146 (34.0)	15 (62.5)	64 (53.3)	<0.001
Time in therapeutic range, %	49.1 ± 28.6	50.9 ± 28.4	48.1 ± 33.3	45.7 ± 27.9	0.527
Time in therapeutic range < 60%	113 (19.7)	68 (15.9)	7 (29.2)	38 (31.7)	<0.001
Direct oral anticoagulants	243 (42.4)	202 (47.1)	7 (29.2)	34 (28.3)	<0.001
Dabigatran	54 (9.4)	48 (11.2)	2 (8.3)	4 (3.3)	0.033
Rivaroxaban	50 (8.7)	37 (8.6)	1 (4.2)	12 (10.0)	0.645
Apixaban	94 (16.4)	75 (17.5)	3 (12.5)	16 (13.3)	0.483
Edoxaban	45 (7.9)	42 (9.8)	1 (4.2)	2 (1.7)	0.011
Off-label reduced dose	67 (11.7)	55 (12.8)	3 (12.5)	9 (7.5)	0.274
Antiplatelet	190 (33.2)	135 (31.5)	8 (33.3)	47 (39.2)	0.285
Aspirin	176 (30.7)	123 (28.7)	7 (29.2)	46 (38.3)	0.126
Thienopyridine	54 (9.4)	43 (10.0)	1 (4.2)	10 (8.3)	0.570
Dual antiplatelet therapy	44 (7.7)	34 (7.9)	0 (0.0)	10 (8.3)	0.349
Pharmacological therapy					
Antiarrhythmic drugs for rhythm control	100 (17.5)	82 (19.1)	2 (8.3)	16 (13.3)	0.164
Class I	79 (13.8)	69 (16.1)	1 (4.2)	9 (7.5)	0.021
Class III	21 (3.7)	13 (3.0)	1 (4.2)	7 (5.8)	0.349
Antiarrhythmic drugs for rate control	312 (54.5)	216 (50.3)	16 (66.7)	80 (66.7)	0.003
Class II	255 (44.5)	175 (40.8)	14 (58.3)	66 (55.0)	0.008
Class IV	89 (15.5)	67 (15.6)	5 (20.8)	17 (14.2)	0.709
Digoxin	60 (10.5)	43 (10.0)	2 (8.3)	15 (12.5)	0.692
Neither of drugs for rhythm or rate control	230 (40.1)	186 (43.4)	7 (29.2)	37 (30.8)	0.025
Non-pharmacological therapy					
History of catheter ablation for AF at baseline	2 (0.3)	2 (0.5)	0 (0.0)	0 (0.0)	0.714
History of pacing device implantation at baseline	10 (1.7)	6 (1.4)	0 (0.0)	4 (3.3)	0.288
Treatment during the observation period					
Electronic cardioversion for AF	22 (3.8)	18 (4.2)	1 (4.2)	3 (2.5)	0.692
Catheter ablation for AF	38 (6.6)	38 (8.9)	0 (0.0)	0 (0.0)	0.001
Maze procedure	6 (1.0)	4 (0.9)	0 (0.0)	2 (1.7)	0.686
Pacemaker implantation	45 (7.9)	33 (7.7)	1 (4.2)	11 (9.2)	0.687
Cardiac resynchronization therapy	5 (0.9)	3 (0.7)	0 (0.0)	2 (1.7)	0.539

TE- and MB-, thromboembolism and major bleeding; M- and HF-, mortality and heart failure; AF, atrial fibrillation.

The prescription rate of antiarrhythmic drugs for rhythm control was comparable among the three clusters (Clusters 1, 16.1%; 2, 4.2%; and 3, 7.5%; P = 0.164). Catheter ablation for AF was performed only in Cluster 1, both at baseline and during the observation period (0.5% and 8.9%, respectively), and no catheter ablation was used in the other clusters (P = 0.714 and P = 0.001, respectively).

## 4 Discussion

### 4.1 Major findings

In the present study, three clusters were identified out of 573 AF patients aged ≥ 75 years from our single-center cohort based on the patterns of the risk scores for mortality (M-score), HF events (HF-score), TE events (TE-score), and MB events (MB-score). Cluster 1 accounted for 75% of the patients, who were characterized as having standard risk scores; Cluster 2 accounted for 4%, who were characterized as having high TE- and MB-scores; and Cluster 3 accounted for 21%, who were characterized as having high M- and HF-scores. The characteristics of each cluster were identified with reference to the patient characteristics and AF-related treatments, including pharmacological and non-pharmacological therapies.

### 4.2 Clinical implications of the cluster analysis in the present study

Cluster analysis can help to identify novel classifications. In a previous report of the use of cluster analysis for AF patients in the ORBIT-AF II registry [7], 9749 AF patients were classified into four clusters, including (1) those with considerably lower rates of risk factors and comorbidities (n = 4673, 48%); (2) those with AF at younger ages and/or with comorbid behavioral disorders (n = 963, 9.9%); (3) those with AF who had similarities to patients with sinus node dysfunction (n = 1651, 16.9%); and (4) those with AF and prior coronary artery disease, myocardial infarction, and/or atherosclerotic comorbidities (n = 2462, 25%). Another report of cluster analysis for AF patients in a Japanese multicenter registry (KiCS) identified three clusters out of 2458 AF patients [8], which included (1) paroxysmal AF in younger people (n = 1190, 48%); (2) persistent/permanent AF with left atrial enlargement (n = 1143, 47%); and (3) atherosclerotic comorbid AF in older adults (n = 125, 5%). Both cluster analyses [7], [8] used multiple patient characteristics at baseline, blinding the patient outcomes. They used AF cohorts that included both young and older adults and commonly identified half of the patients to be at a low risk, characterized by young age, paroxysmal AF, and a low rate of risk factors and comorbidities [7], [8]. Although these cluster analyses clearly identified novel classifications, they did not separate the incidence patterns of AF-related adverse events, including TE-, MB-, and HF events and all-cause death. Moreover, the low-risk cluster, which accounted for approximately half of the total AF patients, was mainly comprised of young patients.

The present study focused on older adults with AF. In young AF patients, symptoms and stroke prevention carry a much higher weight than bleeding or mortality. Inversely, in older adults with AF, AF-related treatment is often delayed because of a fear of bleeding during anticoagulation therapy as well as multimorbidity and polypharmacy, which increase the risk of HF; additionally, aggressive treatment interventions are avoided due to the high potential for iatrogenic adverse events. In such situations, our aim was to prioritize the treatment needs of older adults with AF patients who are at high risk levels for multiple outcomes. Using hierarchical cluster analysis based on computed risk probabilities for representative AF-related outcomes, we adopted a simple approach using three clusters to categorize the older adults with AF patients in our cohort and identify each proportion.

We first identified Cluster 1, which accounted for 75% of the older adults with AF and had mean M-score of 0.04491, mean HF-score of 0.079200, mean TE-score of 0.02239, and mean MB-score of 0.02910. These scores can be translated into incidence probabilities of 4.5% for all-cause death, 7.9% for HF events, 2.2% for TE events, and 2.9% for MB events. In previous observational studies with AF older adults, the incidence rates were reported to be over 3% for TE events, ∼4% for MB events, and over 10% for all-cause death [13], [14]. In clinical trial settings, the incidence rates of stroke or systemic embolism and MB events in older adults with AF patients were approximately 2–2.5% per year and over 4% per year, respectively [15], [16], [17]. Although the incidence rates found in previous cohorts were higher than those for Cluster 1 in our cohort, we regarded Cluster 1 as the “standard risk” cluster because the patient characteristics were generally similar to our overall patient cohort. Compared with the other clusters, the representative clinical features of these patients included a relatively young age (79.4 years old), a higher prevalence of non-valvular AF (92.5%), and a lower prevalence of asymptomatic AF (15.9%) and structural heart diseases (ischemic heart disease, 12.8%; valvular heart disease, 31.5%; cardiomyopathy, 4.2%). Compared to the previous cohorts [13], [14], our cohort included more recently registered patients who were, therefore, prescribed more DOACs. Given that the widespread use of DOACs has contributed to the improved prognosis of AF patients [18], older adults with AF provided DOACs [19], [20] may have more favorable clinical outcomes. As for AF management, more of the patients in Cluster 1 were prescribed class I antiarrhythmic drugs. Moreover, catheter ablation was only performed for the patients in Cluster 1. The proportions of patients in Cluster 1 treated with rhythm control (19.1%), rate control (50.3%), or neither (43.4%) and catheter ablation (8.9%) were mostly comparable to those in a large-scale, nation-wide observational study of Japanese older adults with AF (ANAFIE registry) [21], [22]. These clinical features suggest that common AF-related treatments can be considered for older adults with AF patients who are categorized into Cluster 1.

Second, we identified Cluster 2, which accounted for 4% of the older adults with AF and had a mean M-score of 0.15167, mean HF-score of 0.14127, mean TE-score of 0.14684, and mean MB-score of 0.22601. These scores translate to incidence probabilities of 15% for all-cause death, 14% for HF events, 15% for TE events, and 23% for MB events. The incidence rates of TE and MB events were extremely high, and therefore, we regarded Cluster 2 as having a high risk of TE and MB. Cluster 2 was characterized by a high proportion of patients with HAS-BLED scores ≥ 3 points, a history of ischemic stroke or TIA, and asymptomatic AF. Moreover, the patients in Cluster 2 had a low BMI, low serum albumin, and a high incidence of falls, indicating the existence of frail patients. Notably, irrespective of the high prescription rate of oral anticoagulants (87.5%), the risk of TE was extremely high. Asymptomatic AF is associated with an increased risk of TE [23], presumably because of a low adherence to anticoagulation therapy. In elderly AF patients, a history of bleeding events under anticoagulation therapy can be a significant risk factor for TE events [24], and the unintended discontinuation of anticoagulants would compound their risk for TE. Moreover, the prescription rate of warfarin was higher in Cluster 2 than Cluster 1, with a high proportion of patients having a low TTR (<60%), which may be associated with a high risk of TE and MB. Reflecting the possible existence of frail patients, the prescription rate of antiarrhythmic drugs for rhythm control was low, and no patients underwent catheter ablation. When taken together, the results show that the patients in Cluster 2 would need more aggressive interventions for stroke prevention, and education to increase adherence should be mandatory. Given the high prevalence of a history of both ischemic and hemorrhagic strokes in Cluster 2, DOACs would be a good choice for this group [25]. To prevent cognitive decline, both DOACs [26] and catheter ablation [27] would be helpful. As the patients in Cluster 2 were relatively young, catheter ablation should have been applied more aggressively.

Third, we identified Cluster 3, which accounted for 21% of our elderly AF patients and had mean M-score of 0.20078, mean HF-score of 0.36360, mean TE-score of 0.02394, and mean MB-score of 0.03410. These scores translate to incidence probabilities of 20% for all-cause death, 36% for HF events, 2.4% for TE events, and 3.4% for MB events. Cluster 3 was characterized by a high proportion of older patients with heart failure and structural heart diseases. Moreover, Cluster 3 had a high proportion of patients with HAS-BLED score ≥ 3 points, low BMI, low serum albumin, and a high incidence of falls. In addition, the proportion of those with low eGFR and low CCr was extremely high. It is intriguing that the risks of TE and MB in Cluster 3 were generally similar to those in Cluster 1, while the clinical features in Clusters 3 and 2 were mostly similar in terms of the average CHADS_2_ and HAS-BLED scores, high prescription rate of warfarin, and high prevalence of fall history. Although the reasons are unclear, we speculated that the higher risks of TE and MB were masked by the high frequency of the competing events of all-cause death in this cluster. As for the drugs used for AF management, the prescription rate of antiarrhythmic drugs for rhythm control was low, and no patient underwent catheter ablation, reflecting the possible existence of frail patients, as with Cluster 2. When taken together, our analyses suggest that the patients in Cluster 3 need careful management for AF treatment. Although anticoagulation therapy is essential, the decline in renal function due to increased age and low body weight limits the application of DOACs. For such patients, low-dose DOACs may be the possible choice [28]. Although catheter ablation is beneficial, even in AF patients with heart failure [5], [29], the benefit is limited in elderly patients, patients with longstanding persistent AF, and those with severe heart failure [5], [29].

There were several limitations to this study. First, all the participants were patients who had visited a cardiovascular hospital. Therefore, the results cannot be easily extrapolated to other cohorts, such as general populations. Second, the number of patients was very small; therefore, to obtain a more robust perception, more investigations of larger populations are needed. Third, the classification used in the present study was based on the risk scores for four patient outcomes. Although we used approximately 20 clinical parameters in the development of the risk scores, unknown factors may affect the risks. Forth, the TTR for patients treated with warfarin was low in the present study compared with elderly AF patients described in a previous report who were registered within the same period [19], [20]. This may be because our data included the time period just after warfarin was started. Fifth, if our cluster is used as one of the components of risk scoring systems for application (or not application) of guideline-based treatment, such as catheter ablation [30], [31], [32], [33] or anticoagulation therapy [34], [35], [36], in older AF patients, a caution is necessary. For this purpose, validation of the generality of our cluster will be mandatory.

### 4.3 Conclusion

Based on our cluster analysis, three-quarters of the elderly AF patients in our cohort were at a standard risk and approximately 20% were at a high risk for all-cause death and HF. Notably, the remaining 5% were at a high risk for TE and MB despite the high prescription rate of anticoagulants. The cluster analysis identified those at a high risk for all-cause death and HF or at a high risk for TE and MB, and the data could support decision making for elderly AF patients.

## Author contributions

SS and TY conceived the study concept and study design. SS analyzed the data. All authors collected the data and drafted the manuscript. TY checked the analyzed data and the manuscript. All authors approved the final version.

Acknowledgments of grant support

Dr. Suzuki received research funding from Mitsubishi Tanabe Pharm, and Daiichi Sankyo. Dr. Yamashita has received research funds and/or lecture fees from Daiichi Sankyo, Bayer Yakuhin, Bristol-Myers Squibb, Pfizer, Nippon Boehringer Ingelheim, Eisai, Mitsubishi Tanabe Pharm, Ono Pharmaceutical, and Toa Eiyo. This study was partially supported by the Practical Research Project for Life-Style Related Diseases, including Cardiovascular Diseases and Diabetes Mellitus, from Japan Agency for Medical Research and Development, AMED (JP17ek0210082).

## Declaration of Competing Interest

The authors declare that they have no known competing financial interests or personal relationships that could have appeared to influence the work reported in this paper.

## Appendix A Supplementary material

Supplementary data 1

Supplementary Table 1 Multiple logistic regression models for four patient outcomes.

Supplementary Table 2 Distribution of risk scores in the three clusters.
