# Supplementary material for: Identifying risk patterns in older adults with atrial fibrillation by hierarchical cluster analysis: A retrospective approach based on the risk probability for clinical events
Source: Int J Cardiol Heart Vasc. 2021 Sep 28;37:100883. doi: 10.1016/j.ijcha.2021.100883 (PMC8487977; doi:10.1016/j.ijcha.2021.100883)
Supplement: Supplementary data 1 [file mmc1.docx]

**Supplementary document**

**S1.1. Data collection at initial visit --- p. 1**

**S1.2 Patient follow-up --- p. 2**

**S2. References --- p. 3**

**S1.1. Data collection at initial visit**

After ECG and chest X-ray were performed, cardiovascular status was evaluated using the data of echocardiogram, exercise test, 24-hour Holter recording, and blood laboratory tests at the discretion of the attending physician. In addition to gender, age, height, and weight, we collected data on cardiovascular diseases, including heart failure (New York Heart Association class ≥ 2), valvular heart disease (moderate or severe stenosis or regurgitation on echocardiogram),[1-3] ischemic heart disease (diagnosed on angiogram or scintigram), hypertrophic and dilated cardiomyopathy (diagnosed on echocardiography or magnetic resonance imaging [MRI]), congenital heart disease (diagnosed on echocardiography), and history of disabling ischemic stroke or transient ischemic attack (TIA; diagnosed on computed tomogram or MRI). Cardiovascular risk factors were defined as follows: hypertension, use of antihypertensive agents, systolic blood pressure ≥ 140 mmHg, or diastolic blood pressure ≥ 90 mmHg; diabetes mellitus, use of oral hypoglycemic agents or insulin, or glycosylated hemoglobin ≥ 6.5%; dyslipidemia, use of statin or drugs for lowering triglyceride, low-density lipoprotein ≥ 140 mg/dL, high-density lipoprotein < 40 mg/dL, or triglyceride ≥ 150 mg/dL; and, chronic kidney disease, estimated glomerular filtration rate (eGFR) < 60 mL/min/1.73 m^2^. Body mass index (BMI) was calculated as weight in kilograms divided by height in meters squared. Estimated GFR was calculated using the Japanese coefficient for the modified isotope dilution mass spectrometry-traceable 4-variable Modification of Diet in Renal Disease study equation: eGFR = 194 × SCr^−1.004^ × Age^−0.287^ × 0.739 (if female).[4] Estimated creatinine clearance (CCr) was calculated using the Cockcroft-Gault equation: eCCr = (140 - age) × body weight / (72 × SCr).[5] Charlson's comorbidity index was determined according to the updated criteria reported in 2011.[6]

**S1.2 Patient follow-up**

The health status and the incidences of cardiovascular events and mortality were maintained in the database by being linked to the medical records of the hospital, and by study documents of prognosis sent once per year to those who stopped hospital visits or who were referred to other hospitals. In the present study, we included the follow-up data until March 2020, and excluded follow-up data of >3 years after the initial visit to avoid imbalance of follow-up period among patients due to the different registration years (between 2010 and 2018).

**S2. References**

[1] Zoghbi WA, Adams D, Bonow RO, et al. Recommendations for Noninvasive Evaluation of Native Valvular Regurgitation: A Report from the American Society of Echocardiography Developed in Collaboration with the Society for Cardiovascular Magnetic Resonance. J Am Soc Echocardiogr. 2017;30:303-71.

[2] Baumgartner H, Hung J, Bermejo J, et al. Echocardiographic assessment of valve stenosis: EAE/ASE recommendations for clinical practice. J Am Soc Echocardiogr. 2009;22:1-23; quiz 101-2.

[3] Baumgartner H, Hung J, Bermejo J, et al. Recommendations on the Echocardiographic Assessment of Aortic Valve Stenosis: A Focused Update from the European Association of Cardiovascular Imaging and the American Society of Echocardiography. J Am Soc Echocardiogr. 2017;30:372-92.

[4] Matsuo S, Imai E, Horio M, et al. Revised equations for estimated GFR from serum creatinine in Japan. Am J Kidney Dis. 2009;53:982-92.

[5] Cockcroft DW, Gault MH. Prediction of creatinine clearance from serum creatinine. Nephron. 1976;16:31-41.

[6] Quan H, Li B, Couris CM, et al. Updating and validating the Charlson comorbidity index and score for risk adjustment in hospital discharge abstracts using data from 6 countries. Am J Epidemiol. 2011;173:676-82.
