# Supplementary material for: Identifying risk patterns in older adults with atrial fibrillation by hierarchical cluster analysis: A retrospective approach based on the risk probability for clinical events
Source: Int J Cardiol Heart Vasc. 2021 Sep 28;37:100883. doi: 10.1016/j.ijcha.2021.100883 (PMC8487977; doi:10.1016/j.ijcha.2021.100883)
Supplement: Supplementary Table 1 — Multiple logistic regression models for four patient outcomes. [file mmc2.pdf]

Supplementary Table 1. Multiple logistic regression models for four patient outcomes

	All-cause death		HF events		TE events		MB events	
	OR	P value	OR	P value	OR	P value	OR	P value
Age	1.110	0.008	1.125	<0.001	1.024	0.747	0.989	0.856
Sex (Male:1/Female:0)	1.558	0.232	0.984	0.957	1.723	0.363	2.363	0.125
Body mass index, kg/m^2								
≥25	(Reference)		(Reference)		(Reference)		(Reference)	
18.0-24.9	0.516	0.124	0.892	0.737	0.840	0.786	1.342	0.615
<18.0	0.909	0.887	1.135	0.833	1.250	0.864	4.912	0.100
Systolic blood pressure, mmHg								
≥150	(Reference)		(Reference)		(Reference)		(Reference)	
100-149	0.517	0.146	0.968	0.935	0.317	0.061	1.437	0.612

<100	0.540	0.597	0.430	0.357	0.000	0.998	1.292	0.851
Albumin, g/dL								
≥4.5	(Reference)		(Reference)		(Reference)		(Reference)	
3.5-4.4	2.233	0.450	10.819	0.030	3.991×10^{7}	0.997	7.393×10^{7}	0.997
<3.5	5.047	0.148	14.677	0.019	2.472×10^{7}	0.997	1.731×10^{8}	0.997
Hemoglobin, g/dL								
≥13.0	(Reference)		(Reference)		(Reference)		(Reference)	
11.0-12.9	0.911	0.832	1.018	0.956	1.395	0.603	0.740	0.583
<11.0	2.966	0.037	1.146	0.754	2.617	0.305	1.684	0.500
Estimated creatinine clearance, mL/min								
≥50	(Reference)		(Reference)		(Reference)		(Reference)	
30-49	2.573	0.052	0.953	0.887	0.519	0.346	0.362	0.074
<30	2.086	0.297	0.596	0.376	0.345	0.424	0.000	0.997

Charlson's comorbidity index (updated in 2011)	1.062	0.729	0.915	0.589	1.125	0.678	1.128	0.666
Fall or fracture during the observation period	2.136	0.214	4.006	0.006	2.601	0.355	0.339	0.402
Ischemic heart disease	1.227	0.683	0.580	0.204	0.212	0.187	0.291	0.187
Valvular heart disease	1.483	0.406	2.929	0.010	2.104	0.363	1.770	0.423
Cardiomyopathy	2.416	0.068	3.285	0.002	0.639	0.697	1.920	0.398
Heart failure (NYHA ≥II)	0.345	0.024	3.072	0.001	0.479	0.360	0.778	0.700
Hypertension	0.659	0.319	1.238	0.545	2.194	0.348	1.417	0.536
Dyslipidemia	1.448	0.358	1.438	0.264	2.002	0.250	1.099	0.868
Diabetes mellitus	0.720	0.457	1.775	0.071	0.678	0.593	0.578	0.379
Hyperuricemia	1.323	0.449	1.006	0.985	0.737	0.641	0.408	0.159
History of cerebral infarction or transient ischemic attack	1.622	0.331	1.140	0.770	2.224	0.263	3.471	0.046
History of intracranial	1.575	0.719	2.623	0.408	8.678	0.105	11.286	0.068

hemorrhage								
Chronic obstructive pulmonary disease	11.467	0.002	1.806	0.557	0.000	0.998	0.000	0.998
Maintenance dialysis	1.414	0.718	1.195	0.856	0.000	0.998	3.192×10^{7}	0.997

HF, heart failure; TE, thromboembolism; MB, major bleeding, OR, odds ratio; NYHA, New York Heart Association functional classification.
