# Supplementary material for: Identifying risk patterns in older adults with atrial fibrillation by hierarchical cluster analysis: A retrospective approach based on the risk probability for clinical events
Source: Int J Cardiol Heart Vasc. 2021 Sep 28;37:100883. doi: 10.1016/j.ijcha.2021.100883 (PMC8487977; doi:10.1016/j.ijcha.2021.100883)
Supplement: Supplementary Table 2 — Distribution of risk scores in the three clusters. [file mmc3.pdf]

Supplementary Table 2. Distribution of risk scores in the three clusters

	M-score		HF-score		TE-score		MB-score	
	Mean (SD)	Max /Min	Mean (SD)	Max /Min	Mean (SD)	Max /Min	Mean (SD)	Max /Min
Cluster 1 (Average risk)	0.04491 (0.04396)	0.34714 /0.00359	0.079200 (0.07500)	0.36902 /0.00112	0.02239 (0.03098)	0.25849 /0.00000	0.02910 (0.03018)	0.16402 /0.00000
Cluster 2 (High TE- and MB- risk)	0.15167 (0.17741)	0.71440 /0.01209	0.14127 (0.09958)	0.36346 /0.02403	0.14684 (0.13721)	0.50384 /0.00000	0.22601 (0.09418)	0.50198 /0.11929
Cluster 3 (High M- and HF- risk)	0.20078 (0.16168)	0.77799 /0.00622	0.36360 (0.19975)	0.80755 /0.02383	0.02394 (0.02426)	0.10664 /0.00000	0.03410 (0.04276)	0.17210 /0.00000

HF, heart failure; TE, thromboembolism; MB, major bleeding, SD, standard deviation, M, mortality.
